# Experiences of women who have lost young children to AIDS in KwaZulu-Natal, South Africa: a qualitative study

**DOI:** 10.1186/1758-2652-13-50

**Published:** 2010-12-09

**Authors:** Craig Demmer

**Affiliations:** 1 Lehman College of the City, University of New York, USA

## Abstract

**Background:**

AIDS continues to be the leading cause of death in South Africa. Little is known about the experiences of mothers who have lost a young child to AIDS. The purpose of this qualitative study was to explore the attitudes and experiences of women who had lost a young child to HIV/AIDS in KwaZulu-Natal Province, South Africa.

**Methods:**

In-depth interviews were conducted with 10 women who had lost a child to AIDS. The average age of the deceased children was six years. Interviews were also conducted with 12 key informants to obtain their perspectives on working with women who had lost a child to AIDS. A thematic analysis of the transcripts was performed.

**Results:**

In addition to the pain of losing a child, the women in this study had to endure multiple stresses within a harsh and sometimes hostile environment. Confronted with pervasive stigma and extreme poverty, they had few people they could rely on during their child's sickness and death. They were forced to keep their emotions to themselves since they were not likely to obtain much support from family members or people in the community. Throughout the period of caring for a sick child and watching the child die, they were essentially alone. The demands of caring for their child and subsequent grief, together with daily subsistence worries, took its toll. Key informants struggled to address the needs of these women due to several factors, including scarce resources, lack of training around bereavement issues, reluctance by people in the community to seek help with emotional issues, and poverty.

**Conclusions:**

The present study offers one of the first perspectives on the experiences of mothers who have lost a young child to AIDS. Interventions that are tailored to the local context and address bereavement issues, as well as other issues that affect the daily lives of these mothers, are urgently needed. Further studies are needed to identify factors that promote resilience among these women.

## Background

In 2008, there were approximately two million deaths worldwide from AIDS, including 280,000 deaths among children under 15 years of age. Sub-Saharan Africa remains the most heavily affected region in the world with 1.4 million deaths [1]. In South Africa, which has the largest number of people living with HIV/AIDS in the world, AIDS continues to be the leading cause of death. In 2007, there were approximately 5.7 million people living with HIV in South Africa, and 350,000 people died of AIDS in that year alone [2]. We do not know the cumulative number of children who have died of AIDS in South Africa or even how many children died of AIDS within the past year. In the 2010 country progress report on HIV/AIDS, the South African government acknowledged that the lack of reliable data on infant mortality was a problem [1]. Recent estimates indicate that 2.5% of children aged two to 14 years old are living with HIV in South Africa [3]. For 2009, the total number of new HIV infections in South Africa was estimated to be 413,000, including 59,000 among children [4]. KwaZulu-Natal continues to be the most affected province in South Africa with 37.4% of pregnant women living with HIV [3].

This article describes a study that explored the experiences of mothers in KwaZulu-Natal who had lost a young child to AIDS. The unwillingness of successive governments in South Africa to deal effectively with the AIDS epidemic - most notably the failure to provide access to life-prolonging antiretroviral treatment (ART) to people with HIV/AIDS - has resulted in the needless deaths of many children and adults to AIDS [5]. For the period, 2002-2005, it was calculated that more than 330,000 premature deaths could have been prevented if the government had provided antiretroviral drugs to people with AIDS, and 35,000 babies were born with HIV because nevirapine was not administered to prevent pregnant women from infecting their babies [6].

Since this time, ART has become more widely available to people with HIV/AIDS in South Africa, but there continue to be problems with implementation. For instance, recent estimates indicate that only 70,000 children were receiving ART in 2009 out of 106,000 who needed it [4]. Providing ART to children presents special challenges, including the difficulty of diagnosing HIV in children, faster progression to AIDS and death, and challenges in developing appropriate and affordable ART regimens for children [1]. Furthermore, a significant number of children still have trouble adhering to ART regimens [7].

Considering the high number of AIDS deaths in South Africa, it is disappointing that the issue of AIDS-related bereavement in the South African context has not been adequately addressed [8,9]. Part of the reason may be that there is little discussion in South African society about AIDS deaths or acknowledgement that AIDS was the cause of death when someone has died [10]. Only a handful of studies on AIDS-related bereavement have been conducted in the South African context and they demonstrate the substantial impact that AIDS deaths have had on surviving adults and children [11-13].

## Methods

### Participants

In this qualitative study, two distinct groups were targeted for interviews. The first group consisted of 10 women in KwaZulu-Natal who had lost a child to AIDS. The second group consisted of 12 professionals in KwaZulu-Natal who had experience working with children and families affected by HIV/AIDS. Purposive sampling was used for each group to select participants with a range of experiences [14]. Several local community-based organizations served as gatekeepers for locating potential participants. The use of community gatekeepers is particularly recommended when conducting research with vulnerable families [15].

To be eligible for the first group, participants had to be women who had lost a biological child under the age of 18 years to AIDS. Participants in the second group were selected based on their knowledge and experience of working with children and families impacted by HIV/AIDS in the region. They were professionals employed in local non-governmental organizations, clinics and hospitals in and around the city of Durban and nearby urban area of Pinetown.

### Procedure

Data collection took place from June 2008 to May 2009. Participants in both groups were recruited and interviewed until it was determined that no new themes emerged from the analyses (i.e., a state of theoretical saturation was reached) [16]. To put it another way, the consistency and breadth of themes identified in these interviews suggests that a sufficient number of interviews were conducted to give the analysis depth and relevance. Semi-structured interviews were conducted with participants from both groups. Interviews typically ran for an hour to an hour and a half.

Participants in the first group were asked to describe their experiences of losing a young child to AIDS and how their present lives had been affected by the death of the child. Participants in the second group were asked to describe their experiences of working with mothers who had lost a child to AIDS. All interviews were scheduled at a time and place convenient for participants from both groups and were conducted by trained Zulu-speaking social workers. The importance of establishing trust with participants was reinforced among the trained interviewers, as was the need for the voices of participants to be heard and to be aware of one's own feelings and prejudices [17].

All interviews were conducted in isiZulu and recorded. They were transcribed verbatim and translated into English by the interviewers. The suggestions of Horowitz, Ladden and Moriarity [15] were followed by communicating the relevance of the study to potential participants, making the data collection process as user-friendly as possible, stressing that all views and perspectives were welcomed, and providing appropriate reimbursement to participants. In acknowledgement of their contribution to the study, participants in the first group were paid R70 (approximately $10) after completing the interview. Professionals in the second group received no compensation for their participation in this study. Being mindful that the interview process could be stressful or even traumatic for participants (in both study groups), the interviewers solicited feedback from participants about how they were feeling as a result of the interview, and referrals to local mental health resources were made when needed.

### Data analysis

A thematic analysis was employed in this study. Transcripts of the interviews were carefully read, and patterns and themes were identified. Following the coding procedures outlined by Strauss and Corbin [16], phenomena were grouped into categories of like meaning and the contents of the categories were compared between and within interviews. There was a continuous process of collecting data and comparing data with previously coded data. The qualitative software programme, NVivo8, was used to mechanically code and facilitate the analysis of the transcripts of the in-depth interviews. Software programmes such as this facilitate hierarchical or "tree-like" coding and analysis of large amounts of text across multiple themes [18]. To ensure trustworthiness [19], the research team discussed coding, themes and key findings until consensus was reached.

### Ethical considerations

This study was approved by the institutional review boards of the University of KwaZulu-Natal in South Africa and Lehman College in the United States. Bearing in mind that some participants in the first group (mothers) could be illiterate or have poor reading comprehension, as well as the fact that there was a risk of these participants' names being linked to consent forms in a country with high HIV stigma, oral informed consent was obtained from these participants, instead of written informed consent. Prior to each interview, the interviewer followed a prepared script (written in isiZulu), which they read to each participant advising them of the purpose of the study and requesting their permission to audiotape the interview. Participants were advised that whatever they said would be kept confidential and that they were free to stop participating at any time. Written, informed consent was obtained before each interview from participants in the second group.

## Results

Participants from the first group (mothers) ranged in age from 25 to 60 years and the mean age was 30. All were HIV-positive, black women who lived in the province of KwaZulu-Natal. Two were married, two were widowed, four had partners, and two were single. One-half of participants were receiving ART. One participant had completed high school and the rest had completed only a few years of primary schooling. One participant had a temporary full-time job and one had a part-time job; the other participants were unemployed and had not been able to find formal employment in several years.

Two participants lived in their own homes and eight stayed with relatives. The number of people living in each household ranged from four to 13 with an average of six people in each household. Most participants depended on child support grants or old age pensions from grandmothers. They sometimes received financial support from other family members or partners, but this was sporadic. Participants had, on average, two surviving children. The deceased children were, on average, six years old at the time of death, and the average time since the death of the child was two years prior to the study. All participants had lost one child to AIDS, except one participant who had lost two children: a one year old and a four year old.

In the second group (key informants), two participants were male and 10 were female, and their average age was 44. Six participants were counsellors, four were nurses, one was a social worker and one was an AIDS project coordinator. They had, on average, six years of professional experience working with children and families impacted by HIV/AIDS.

Six main themes emerged from the data analysis and are described in the pages that follow: caring for a sick child; the moment of death; relationships with health professionals; daily stresses; coping; and support.

### Caring for a sick child

Some women reported that their child was ill for a very short period of time and then died. This mostly occurred among infants. A few weeks or months after giving birth, the woman would notice that her child was not looking well, the child would be hospitalized, and then die a few days later:

There were no signs at all that she was going to die. She was growing up very well like a normal baby. She was just attacked by the flu. I took her to the hospital and it was the end of her. She died. [Participant 3]

Not all women in this study were aware of their own HIV status before they gave birth. Some had not returned for their HIV results after been tested at the antenatal clinic out of fear that they could be HIV positive. Some women minimized or overlooked symptoms in their child and only sought medical care for the child when the disease was in its advanced stages. Among the key informants interviewed there was a great deal of frustration that the stigma and secrecy surrounding AIDS in South African society continued to cause many needless deaths among children:

They just pretend as if everything is normal. They don't test their children after giving birth. They just pray for miracles to happen, no matter how you educated them on the importance of testing their children at birth. They take action when the child is sick. In most cases, that is too late. [Key informant 4]

Several key informants advocated mandatory HIV testing of all children as a solution to the problem of parents not testing their child or testing the child too late due to stigma. The other consequence of stigma mentioned was parents' failure to ensure that their children adhered to treatment, sometimes resulting in the death of the child.

Not all family members are made part of the caring of the child ... Some parents or caregivers are too preoccupied with hiding the fact that the child is sick and cannot therefore fully comply with support services and other available resources of a sick child. [Key informant 3]

But there were women who knew their HIV status and the cause of their child's illness, and they did everything they could to get their child appropriate medical care, as challenging as it was. Frequent hospitalizations and watching the child's health deteriorate took its toll on these women:

What stressed me so much is that I did not know how to help her. To see her in pain was the most painful thing to me. [Participant 5]

Their sense of helplessness was aggravated by guilt for causing the child's sickness, as well as feeling unsupported during this stressful time. Typically, the only person they could rely on for any kind of help was their own mother. The child's father was usually absent from the picture, either because he was dead or he had abandoned the mother and child. This caused anger and resentment among the women:

The father of these babies are not supportive ... they have children all over but they do not bother about caring for their children. Their children are like mushrooms, I am telling you ... I am not sure whether they even realize the pain that they cause us and the suffering they cause to the children. [Participant 1]

Having little to no support from the child's father, the women struggled to make ends meet each day and worries about money created another layer of stress and impacted their ability to care for the child, especially during long hospital stays:

When my daughter was sick, I was the breadwinner. Even though she was sick, I had to leave her alone and go and sell vegetables so that we can have food on the table. I did not have anyone to help her. I used to think that if there someone at home working and bringing in income, I was going to provide better care to my daughter. I was going to stay at home and look after her. That is what makes me feel guilty and have lots of regrets. I did not provide better care. [Participant 1]

A few women had no-one to help them either emotionally or financially during this period, and talking about it brought back strong memories of being overwhelmed. Some were philosophical about going it alone and had no expectations of receiving support from anyone, but for others, the feeling of rejection still hurt:

Ay sister, I suffered. No-one from my own family and from my child's family supported me (crying). [Participant 2]

### The moment of death

Dredging up memories of the last moments with their child was very painful, yet the women proceeded to describe in detail the moment their child died. All of the children died in the hospital and all women, except one, were present at the time of death. In most cases, the woman was alone with no other family members present. The initial reaction to the death of the child was one of confusion, shock and disbelief. The following statement illustrates a mother's final moments with her child:

She passed away in front of my eyes; then they quickly asked me to leave the ward. I was not prepared to deal with it. My mind was still telling me that maybe the doctors were still going to do something to revive her. I was confused. She managed to calm me down. She died in front of my eyes in hospital ... That is the day I will always remember. It is still fresh as yesterday. [Participant 1]

None of the women talked about being allowed to spend time in the room with their deceased child. The goal of the medical staff seemed to be on removing the mother from the room and taking her elsewhere to calm her down. While some women recalled a social worker or counsellor speaking to them briefly after their child died, this was not usually the case. Typically, the woman called a family member to have them pick her up or she left the hospital alone shortly after the child died.

### Relationships with health professionals

The women reported mixed experiences dealing with doctors and nurses on medical appointments and when their child was in the hospital. Sometimes the quality of care they received depended on a particular shift or the hospital they went to. Some women remembered incidents that still made them angry. A common complaint was being required to sit with their child for long periods of time at the hospital while waiting to be seen by a doctor and being ignored even when the child was clearly in distress:

We were sitting with the child in the hospital benches ... He was not offered even a bed ... The child could not even sit up straight. He was vomiting and had diarrhoea at the same time. He also had stroke in his one side. They only put him on a drip. We called for their attention when the drip was finished. No one cared or responded. The child was bleeding. I said, "You see, this is a waste of time." At 12 we left for home. We took the drip off the child on our way home. [Participant 8]

After the child was admitted to the hospital, the quality of care was a problem for some women. Nurses did not change feeding tubes or did not bath their child, and it was often left to the mother to perform these tasks. Several women exclaimed that nurses "just sit there" and that the only time the nurses responded was when the mothers complained. Not being told what was happening to their child caused frustration and heightened their anxiety. Several women took their child out of the hospital and returned home because they were so dissatisfied with the treatment and the attitude of the medical staff. One woman related this incident:

The doctor did not treat my child's situation as an emergency. She was supposed to hurry up. When she arrived, she ... was supposed to be caring and supportive. Do you know that during the time when my baby was vomiting blood, the doctors ordered me to carry her? I carried her and blood was coming from everywhere. Ay, no one helped me. When I was calling nurses, they were ignoring me. No one helped me. [Participant 5]

Conversely, some women were satisfied and grateful for the way the medical staff treated them and their child. There was recognition of how overworked the medical staff was, and it meant a lot to them when nurses kept them updated on the progress of their child or found time to comfort them. But overall, communication, or rather a lack thereof, by medical staff was a common complaint; it made the women feel both powerless and disrespected. In addition, little to no support or counselling was provided by social workers or counsellors at the hospital, either before the child died or after the death.

Key informants in this study expressed a deep commitment to their work and clearly understood the context in which these mothers lived and the challenges facing them. The issue of scarce institutional resources was a common concern among key informants, as well as the lack of a coordinated response in addressing the needs of these mothers. The issue of grief was frequently not addressed by service providers because these mothers were primarily concerned about meeting urgent daily needs, such as food and shelter, and also because service providers sometimes felt ill prepared to provide this type of counselling. Key informants acknowledged the importance of helping these mothers to open up and to talk about their loss while simultaneously helping them with their daily needs. They expressed some frustration that these mothers were not aware that they needed to take care of their psychological needs as well:

These parents need to acknowledge their pains. They need to talk about their loss. When their children are sick, they need both material and emotional support. [Key informant 6]

### Daily stresses

Besides the trauma of losing a child, these women were confronted with circumstances that compounded and sometimes overshadowed their grief. Most of the women had experienced periods of being very ill and a few had nearly died. The death of a child reminded them of their own mortality. They worried less about dying and more about what would happen to their surviving children if they died. But most of them tried not to think about it and preferred to stay focused on the present and caring for their surviving children:

If I think about death now, I won't reach where I want to be. [Participant 4]

Some women had given birth to another child since their loss and now worried about this child's health, while some were dealing with other children who were HIV positive and sick. Some women had not had their other children tested for HIV or were too afraid to return for the results.

It was difficult for the women to talk to their children about the loss of their sibling, especially that their sibling died of AIDS. Most women were waiting for the right time to talk to their older children about this, but admitted that they were procrastinating and felt that they did not know how to do it. While their children were aware that their mother and sibling had been sick, they were not told it was HIV related.

AIDS-related deaths, both within the family and among people they knew in their local community, were common, yet the nature of the death was rarely acknowledged or discussed because of stigma. Most women had trouble estimating the number of funerals they had attended in recent years for people who were known or suspected to have had AIDS. They made it a point to no longer attend funerals, except for family funerals, and tried to put funerals and death out of their minds. When asked if they knew of other mothers who had lost a child to AIDS, most replied that they did not because it was not talked about.

Problems relating to other family members were a source of great concern. Most women had a partner who was usually the father of one of their children, but most of the time the partner lived elsewhere and they complained about him having multiple girlfriends and neglecting them. Besides not providing them with enough money to care for themselves and their children, these women also had to deal with such issues as alcoholism, domestic violence, and sexual coercion by their partners.

But the most dominant daily worry of these bereaved mothers involved being impoverished: finding money for food, shelter, school fees and so on. Typically, they had few sources of income and they relied on their husbands or boyfriends, as well as their mothers, for money. A couple of women earned a little money selling beads or produce or working part-time as a domestic worker. Jobs were scarce and a lack of education and skills, together with health problems, meant that most women had been unemployed for many years. The only regular source of income was a small monthly government grant that some women received for a sick child, or they relied on a grandmother's old age pension. Households typically consisted of five to 13 people, and the woman and her children lived in a relative's house. Food was scarce and households were simply unable to support all their members. The following statements illustrate the burden of poverty:

In my family we are very poor ... Food is not always available. We sometimes sleep without food. Even my baby goes without food if I have no money to help her. [Participant 3]

Ay, my life is just full of problems ... Whatever money I get, I have to try to meet all my kid's needs and food. There is nowhere to take a cent from. When I buy a two litre cool drink for my kids, they become happy for that day and I become happy with them. I tell them that things will be better in future and I will be a good parent ... I have faith because my partner still provides us with porridge. [Participant 8]

### Coping

The loss of a child was devastating and some women did not want to go on living. It was only the fact that they had other children to worry about that prevented them from giving up. After the child's death, the women assumed responsibility for the burial of the child, sometimes relying on their mothers or another family member to help with the expenses. In only a few cases, the father of the child helped with funeral arrangements or expenses. In several cases, the father did not even attend their child's funeral.

Almost without exception, the women kept their grief to themselves, not because of an unwillingness or inability to confront their loss, but more as a matter of survival. Because of the context in which they lived, the women had no choice but to contain their grief and focus their emotional and physical energy on coping with the hardships of daily life. When asked how they were feeling during the interview, the reaction was typically that talking about their loss reminded them of what happened in the past. They acknowledged that past efforts to suppress their grief were not always successful. No matter how hard they tried to forget and no matter how long ago their child died, certain things would remind them of their loss, and the pain would return.

Despite their best efforts to continue, the women felt overwhelmed by sadness and despair and they no longer felt they were the same person. They felt that they were more irritable and short-tempered, had problems sleeping, and except for their children, they derived little happiness out of life. They were worn down by negative attitudes in society toward people with HIV/AIDS and they felt unloved. Life had not turned out the way they had hoped and there was little chance things would improve in the future for them. One woman carried her daughter's death certificate around in her bag, even though her daughter had died two years previously. She did so to remind herself of her pain. The following statement illustrates the way many of these bereaved mothers felt:

I cannot focus on the past. I have to find a way to manage these feelings when they come ... Even now her space is still there. Her death is still fresh in my mind and heart. It left an empty hole (sobbing). [Participant 1]

African cultural tradition prescribes that when a child dies, the family and community rally around the mother for a few weeks, sitting with her, bringing food, cleaning her house, in addition to talking about the pain of the loss. And supposedly by the time of the funeral, family members have talked enough about the death and are ready to let go of the deceased child. Several key informants mentioned the urge of mothers to move on after the death. Times have changed and key informants acknowledged that communities were no longer tight knit and members often did not know one another. Consequently, there was a greater need for mothers to turn to outsiders for assistance with their grief. Key informants stressed the importance of providing opportunities for the mother to talk about her loss and to not hold it in.

### Support

The women in this study perceived there to be few sources of support available to assist them with their loss. Some could not identify a single person that they could talk to about their loss. They harboured no expectations of their families in this regard. In most cases, they could only talk to their mothers, but even then, AIDS was often circumvented. Some women derived strength from prayer and obtained some measure of support being with other members of their church, but they seldom disclosed their status or revealed the cause of their child's death to church members for fear of being ostracized.

The value of talking about one's feelings was recognized by some women and when asked if they would be interested in joining a support group for mothers who had lost a child to AIDS if it were to be created, most expressed interest. They looked forward to the opportunity of talking to other women who were in similar situations and they felt it would make them feel less alone. The idea of making friends was appealing, but some reservations were expressed about confidentiality and there were concerns about the cost of transport. As one mother explained, "I sometimes can't even afford a loaf of bread." From the mothers' perspective, financial concerns took priority over the need for counselling. They desperately needed help with food, clothing, school fees and caring for their children.

Key informants acknowledged that interventions needed to incorporate both financial and emotional elements and a suitable option would be an income-generation project that provided mothers with the opportunity to share their loss with other mothers while working on something like beadwork or a vegetable garden to earn income:

Most of our clients are unemployed, come from poor families. We felt that we cannot exclusively meet their emotional needs without material assistance. We have created an environment where we can talk about their problems and at the same time attending to their pressing bread and butter needs. [Key informant 6]

Key informants believed that it was important for these mothers to receive emotional or psychological support, but that mothers were frequently unaware of services available. Mothers should be provided with money for transport costs, as well as offered food, since they often travelled long distances on empty stomachs to the organization. Key informants felt that they needed to do a better job of educating mothers about the benefits of receiving counselling and support, as well as making these services more accessible, but a lack of organizational funds and a shortage of mental health professionals made it difficult to do so. The following statements highlight the views of key informants with regard to providing counselling to these mothers:

People like me and you understand the importance of counselling but the people that our organization deals with ... the majority ... are poor and uneducated people who worry more about the physiological needs than counselling. [Key informant 4]

They don't recognize the importance of attending to the emotional self. They place most emphasis on the physical self and neglect the emotional self ... They normally come to our agency for poverty-related conditions. During the conversation you then learn about a sick child or a deceased child ... I think people need to realize that they need to tell us how they want to be helped. We cannot help them if we don't how they want to be helped. [Key informant 6]

## Discussion

This study represents the first known study about the bereavement experiences of women who have lost a young child to AIDS in South Africa. The issue of HIV stigma had a profound influence on the bereavement experiences and daily lives of the women in this study. People with HIV/AIDS have been stigmatized worldwide since the beginning of the epidemic. HIV stigma remains a major obstacle to prevention, treatment and support efforts for people affected by HIV/AIDS in South Africa [20]. In the present study, societal hostility toward people with HIV/AIDS caused the women to delay getting themselves or their babies tested, to not seek out medical treatment for their child in a timely manner, and to not get the support they needed to cope with their child's illness and subsequent death.

Based on these women's experiences, it is evident that as a society, South Africa has still a long way to go to effectively address HIV stigma and to reduce its impact on all aspects of the daily lives of people affected by HIV/AIDS. A study of women living with HIV/AIDS in the Western Cape Province of South Africa showed a relationship between those who experienced more HIV stigma and severe post-traumatic stress, depression, a low quality of life and fear of disclosure [21]. A study of older adults in the Eastern Cape Province of South Africa reported high levels of grief associated with the death of children and/or grandchildren to AIDS, with stigma being the most important predictor of grief [22].

Getting through each day was a challenge for the women in this study. They had to fend for themselves for the most part since there were few people they could count on for support, whether it be financial or emotional support. Families impacted by AIDS deaths in South Africa could benefit from making greater use of mental health services. At the same time, there exist barriers to seeking this type of help, such as stigma, lack of awareness of resources available and the perception by people that physiological needs and issues of daily survival take priority over mental health needs. In a study among residents in a South African township, additional barriers that were cited in terms of using mental health resources were mistrust of mental health professionals, doubts about the nature and value of psychotherapy, and concerns about the ability of professionals to be culturally sensitive [23].

The emotional and physical burden on these bereaved mothers warrants further investigation, especially in light of their HIV status and associated health issues. The evidence suggests that bereavement distress is likely to be greater among HIV-infected individuals than non-infected individuals [24,25]. Furthermore, grief reaction may be more severe among HIV-infected bereaved individuals who are sicker [26]. As a result of the absence of social support and faced with urgent problems related to their socio-economic circumstances, the women in this study spent most of the time repressing their grief. This did not mean that they did not have moments of extreme sadness, but they felt that they had no choice but to put aside their grief so they could have the strength to deal with the hardships of daily life.

There has been growing support in recent years for the view that most people are resilient and are naturally able to cope with the most adverse circumstances without experiencing a significant disruption in daily functioning or requiring psychotherapy [27]. Resilience is defined as "the ability of adults in otherwise normal circumstances who are exposed to an isolated and potentially highly disruptive event, such as the death of a close relation or a violent or life-threatening situation, to maintain relatively stable, healthy levels of psychological and physical functioning" [28]. It would be useful for future studies to measure the extent of resilience among these women and, more importantly, to examine which factors promote resilience among them.

An important gap in our knowledge is how resilience varies across cultures. Using the concept of resilience for examining and understanding the experiences of women in Africa who have lost a child to AIDS would also help us learn how cultures other than those in western countries effectively (and perhaps ineffectively) cope with extreme adversity. We are still learning about what factors promote resilience, but preliminary evidence shows that resilience tends to occur in people who have the personality traits of hardiness and self-enhancement, those who are repressive copers and those who are able to express positive emotion and laughter [29].

In the present study, these women certainly displayed enormous strength and courage in confronting very challenging circumstances and they were able to express positive emotions and possess hope for the future, and it would appear that they possessed qualities consistent with those who have hardy personalities. There was evidence of repressive coping, but it is unclear to what extent this was natural emotional response and to what extent it was the only way these women could cope in light of the absence of support and the need to address more urgent daily needs of survival. These women did not appear to display extraordinary high levels of self-esteem, narcissism or an unrealistic sense of their strengths, which have been associated with the self-enhancement trait [28]. But they also did not dwell on their personal limitations and in a sense, they assumed the role of superwoman because there was no alternative. No-one was coming to their rescue and they had to rely on themselves to deal with their loss and to meet the needs of their surviving children. Future research on resilience needs to more accurately assess the pathways to resilience, especially in contexts that are vastly different to countries in the west.

In this study, mothers assumed total responsibility for their sick child while the father was excused of all responsibility by virtue of having abandoned his family. This is a problem that is endemic in South African society. Throughout the epidemic in South Africa, the burden has fallen on women to be involved in HIV prevention, treatment and support efforts. Men are very difficult to reach and to get involved in community initiatives around HIV/AIDS. Many men have eschewed responsibility in transmitting the virus and for caring for loved ones who are sick. To a large extent, cultural norms have encouraged this. It is essential that men begin assuming more responsibility in the care and support of people with HIV/AIDS, and interventions need to be developed that clearly involve men in these activities [30].

The women in this study reported both positive and negative experiences with healthcare professionals during the time their child was sick in the hospital. A study of patient and provider perceptions in public health clinics in the KwaZulu-Natal and Gauteng provinces in South Africa revealed that there were gaps in HIV/AIDS knowledge among healthcare providers, and patients reported mixed experiences with the quality of care [31]. What was especially troubling in the current study was the minimal amount of support given to these women by hospital staff at the time of the child's death or after the death. Referrals were seldom made to community resources to help these women with their grief or to help them with practical matters, such as funeral arrangements. In essence, many of the mothers felt abandoned by the hospital.

## Conclusions

With the recent push to increase access to ART for people with HIV/AIDS in South Africa, the outlook for HIV-positive mothers and their children is more promising than ever, and hopefully, we will soon see a sharp decline in AIDS deaths. In the meantime, the needs of these women and children deserve top priority and medical treatment needs to be combined with appropriate psychosocial support.

## Competing interests

The authors declare that they have no competing interests.

## Authors' contributions

This manuscript was conceived, drafted and authored by CD.
